# Polymers as the Primary Fabrication Material for Soft Robotic Grippers: A Concise Review

**DOI:** 10.3390/polym17182464

**Published:** 2025-09-11

**Authors:** Maryna Yeromina, Ján Duplák, Samuel Mikuláško, Rastislav Kaščák

**Affiliations:** Department of Manufacturing Technologies, Faculty of Manufacturing Technologies, Technical University of Košice, Bayerova 1, 08001 Prešov, Slovakia; maryna.yeromina@tuke.sk (M.Y.);

**Keywords:** polymers, robotic, gripper, SMPs, dental production

## Abstract

Each component implemented in a robotic system requires a priority on low weight, flexibility, and durability. These key parameters for creating a stable yet flexible device can be achieved by applying polymer materials in the construction, not only in the static parts of the skeleton but also in the design and production of robotic grippers. This concise review highlights the possibilities of using polymers as the primary fabrication material for the specific area of “soft grippers” used in dental production. The main part of the article is devoted to analyzing polymer materials—specifically SMPs—their modifications, the characteristics of their primary properties, and the specifics of their use in robotic grippers. The article also provides information on the current state of research conducted over the last decade, through a brief overview obtained from the renowned Web of Science and Scopus databases. As part of the overview, we used basic keywords relevant to the analyzed issue: soft, gripper, and polymer. The review results indicate continuous growth in the number of publications, with the highest increase recorded in 2016 compared to 2016, showing more than a fourfold increase. The lowest increase was recorded in 2020 compared to 2019, where the publication rate was stagnant. The most frequently published documents are Articles, comprising 80.1% to 68.3% (more than 5000 publications). The most popular categories in which documents were published are Engineering 27.1%, (more than 4500 publications) and Materials Science Multidisciplinary 45.2% (more than 180 publications). Based on this review, the importance and need for research into this issue and its analysis can be confirmed, not only in practical applications but also within theoretical reviews and summaries.

## 1. Introduction

**Figure 1:**
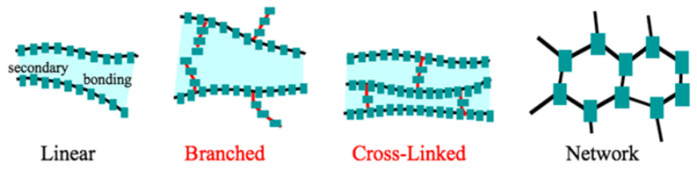
Structure of polymers macromolecules [2].

Polymers are high-molecular-weight compounds formed by the chemical bonding of many repeating structural units called monomers, most commonly through polymerization reactions. These macromolecules can be of natural origin—such as cellulose, starch, proteins, or DNA—or synthetically produced, for example, polyethylene, polypropylene, nylon, or PVC [1]. Polymers can exhibit linear, branched, or cross-linked architectures, which significantly influence their physical and chemical properties, including elasticity, strength, thermal resistance, and solubility. They are categorized into thermoplastics, which can be repeatedly reshaped upon heating, and thermosets, which retain a fixed structure once cured. Polymers are widely utilized across various sectors, including industry, construction, healthcare, and food packaging, and are present in plastics, textiles, adhesives, and medical devices. Research and development in polymer science play a crucial role in advancing modern technologies and promoting sustainable development. The properties of polymers, in addition to the value of the average molar mass, also depend on the molecular and supramolecular structure of the polymers. Macromolecules can be divided into five types based on their structure (see Figure 1) [2].

Linear polymers represent a very widespread group, consisting of monomers connected end-to-end with neighboring units, forming a linear or chain-like structure. The properties of these polymers are largely determined by the mobility (or flexibility) of their molecular chains. This is because the intermolecular forces between polymer chains are much weaker than the strong covalent bonds within the chains themselves. As a result, these materials exhibit low mechanical strength, can become highly elastic when heated (as in the case of thermoplastics), and are capable of dissolving in certain solvents [3]. The longer the macromolecules and the greater the polarity of the individual links, the higher the strength and hardness of the polymer. Due to their mobility, long macromolecules can form a chaotic, complex system of tangled fibers. Such entanglement of macromolecules hinders their ability to move freely in space, reducing the flow of polymers. Polymers with a linear structure can orient themselves and form crystals. The degree of crystallization depends on the material and the processing method. Polymer strength and hardness increase with macromolecular chain length and the polarity of repeating units. Long chains are flexible and tend to entangle, restricting mobility and flow. Linear polymers can align and crystallize, with crystallinity influenced by material type and processing conditions.

**Figure 2:**
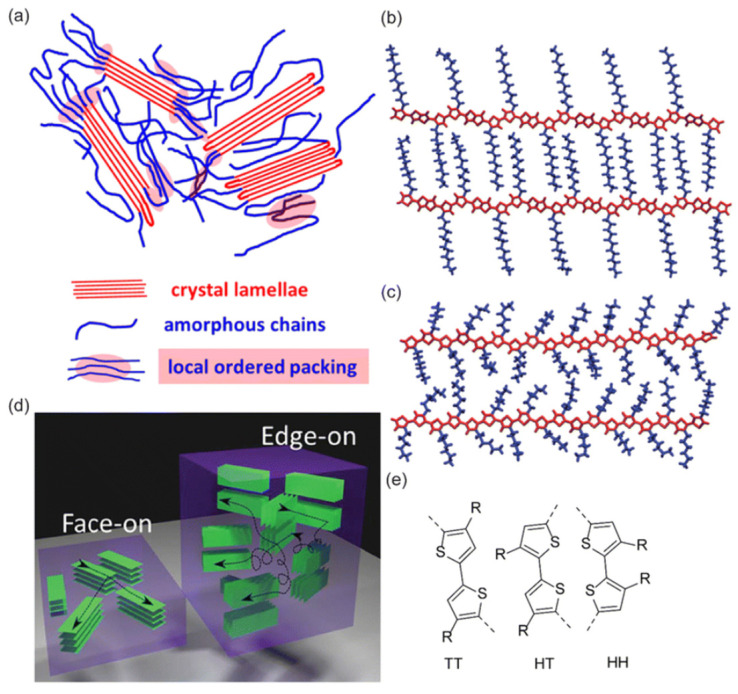
The lamellae of crystalline structures (**a**) crystalline lamellae (red), amorphous chains (blue), and locally ordered packing (pink); (**b**) ordered chain packing in crystalline domains; (**c**) partially disordered packing of polymer chains; (**d**) orientation of crystalline domains with respect to the substrate (*face-on* and *edge-on*); (**e**) structural motifs of chain connectivity: tail-to-tail (TT), head-to-tail (HT), and head-to-head (HH) [3].

Polymers with three-dimensional crosslinked networks from monomers having more than two reactive sites, creating thermosetting plastics. These materials soften minimally on heating and decompose before melting. Higher crosslink density enhances hardness, strength, and thermal stability while reducing solubility, enabling shape retention unlike other polymers [4]. Branched and crosslinked polymers exhibit greater spatial order than linear polymers. Branched polymer properties depend on the length, density, and composition of side chains. Dense branching disrupts macromolecular packing, weakening intermolecular forces, which increases flexibility, cold creep, and plasticity but decreases strength. Linear polymers can be chemically converted into crosslinked networks—known as hardening in plastics, vulcanization in rubber, and drying in coatings [5]. High-molecular-weight substances are composed of macromolecules made of many identical units joined by covalent bonds. The fundamental supramolecular unit is a bundle of macromolecules, which may adopt different conformations. A more organized form of these bundles is the lamellae, which serve as basic elements of crystalline structures (see Figure 2) [2,6].

Semicrystalline polymers consist of tightly packed macromolecules arranged in a regular lattice. However, they always contain both crystalline and amorphous (non-ordered) regions, existing as a two-phase system. Even fully random polymer structures are more ordered than liquids. The degree of crystallinity—typically between 10% and 90%—measures the ratio of crystalline to amorphous phases. Crystalline regions, called crystallites, have molecules arranged in three dimensions but lack the sharp edges seen in low-molecular-weight crystals [7]. These polymers are considered polycrystalline materials, with crystallites forming rigid centers that strongly influence mechanical properties. Phase changes in polymers include transitions from liquid to solid or amorphous to crystalline states. When a molten polymer cools without crystallizing, it forms a hard, glass-like solid called the glassy state. The temperature where this occurs is the glass transition temperature (Tg), marking a sudden change without forming a new phase [8].

**Figure 3:**
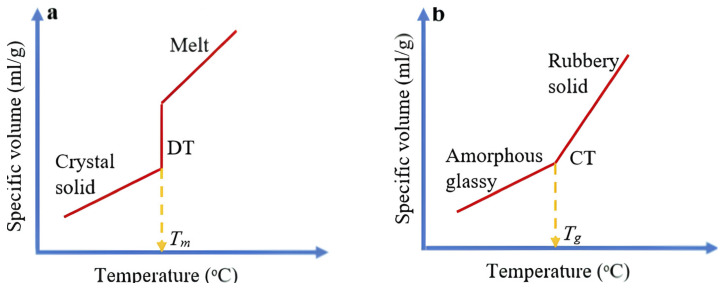
Melting and glass transition temperatures of partially crystalline polymers; (**a**) Transition during melting of a crystalline solid: (1) Hard; (2) Melted. (**b**) Vitrification of an amorphous solid: (3) Glassy; (4) Elastic. Adapted from: Belostotsky, V. Thermodynamic approach to liquid-to-glass transformation as an arrest transition in polydisperse solution. arXiv preprint arXiv:2209.06174, 2022 [10]. Licensed under CC BY 4.0. The figure was redrawn and modified based on the original graphical data.

The glassy state is characterized by features that distinguish it from the melt, including a reduced temperature coefficient, changes in mechanical, optical, thermal, and electrical properties, high viscosity, and variations in specific gravity [9]. A typical curve showing the dependence of specific gravity on temperature is illustrated in Figure 3.

The glassy state in polymers results from reduced mobility—molecular segments essentially “freeze.” At high temperatures, molecular motion is fast, but at low temperatures it slows so much that equilibrium cannot be reached, leaving the polymer in a non-equilibrium glassy state. In this state, volume, enthalpy, and entropy are higher than in the unfrozen melt at the same temperature. The polymer gradually moves toward equilibrium, lowering these values, a process linked to the glass transition temperature (Tg). Above Tg, polymer chains can move due to thermal energy; below Tg, segment mobility decreases, affecting mechanical properties. These changes mainly affect the amorphous parts of crystalline polymers. Transitioning from partially crystalline to amorphous states alters physical, structural, and thermodynamic properties, including volume and heat content. Because polymer chains span both phases without a sharp interface, the amorphous-crystalline boundary is diffuse. This disorder raises surface energy and broadens the melting range [9]. Shape memory polymers can be activated by various external stimuli that trigger their transition between a temporary and a permanent shape. The most used stimuli include heat (thermal activation), light (photothermal activation), electric fields, magnetic fields, or changes in pH or humidity. The choice of stimulus depends on the specific application and the desired material properties.

**Figure 4:**
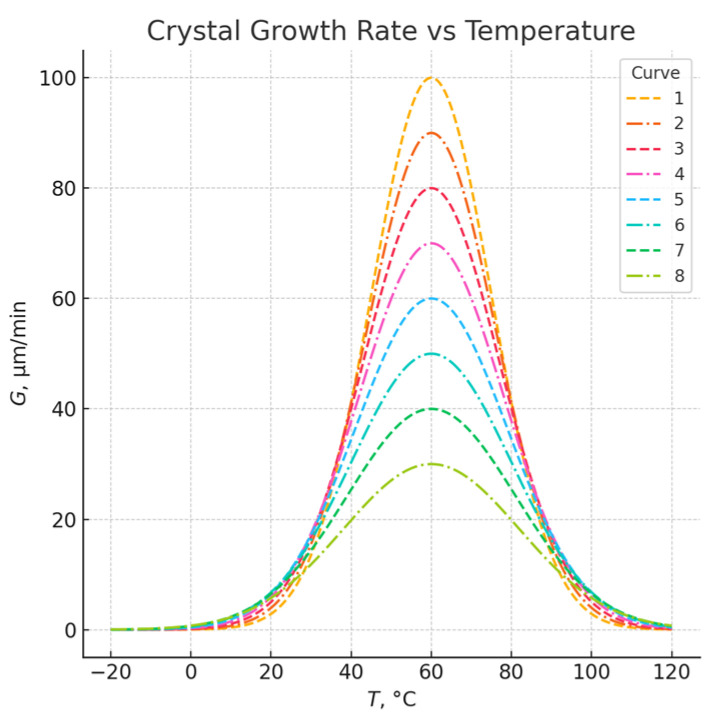
Crystallization rate as a function of temperature for semicrystalline polymers. Molecular weight: (1) 8700 g·mol^−1^; (2) 10,000 g·mol^−1^; (3) 15,800 g·mol^−1^; (4) 25,000 g·mol^−1^; (6) 37,500 g·mol^−1^; (7) 56,000 g·mol^−1^; (8) 143,000 g·mol^−1^. Adapted from: Gabana, Kutlwano, Gehring, G.A., Zeng, X., and Ungar, G. Quantitative model of multiple crystal growth rate minima in polymers with regularly spaced substituent groups. *Macromolecules*, **2024**, vol. 57, no. 4, pp. 1667–1676 [12]. Licensed under CC BY 4.0. Available from: https://doi.org/10.1021/acs.macromol.3c02432.

For a partially crystalline polymer, the melting temperature of the crystalline regions (Tm) and the glass transition temperature (Tg) are shown in Figure 4. The melting temperature Tm is typically about 200 °C higher than Tg. Crystallization starts when the polymer cools below Tm and continues over a temperature range down to Tg. The crystallization rate strongly depends on temperature [11]. Such a chart is important for optimizing processing conditions (e.g., molding temperatures, cooling) in injection molding, 3D printing, or extrusion, where crystallinity control affects the mechanical properties and stability of the final product.

**Figure 5:**
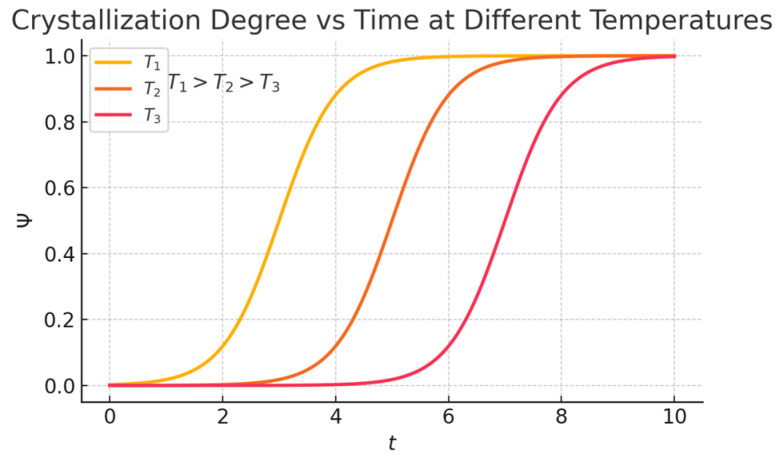
Isothermal crystallization of polymers. T_1_, T_2_, T_3_—induction periods. Adapted from: Adapted from: Gabana, Kutlwano, Gehring, G.A., Zeng, X., and Ungar, G. Quantitative model of multiple crystal growth rate minima in polymers with regularly spaced substituent groups. *Macromolecules*, **2024**, vol. 57, no. 4, pp. 1667–1676 [12]. Licensed under CC BY 4.0. Available from: https://doi.org/10.1021/acs.macromol.3c02432.

Isothermal crystallization of polymers is illustrated in Figure 5. The process begins with a slow increase in crystalline, which then transitions into a rapid growth phase, eventually approaching an apparent equilibrium.

When crystallization time is extended, crystallites grow in a stepped manner. Primary crystallization forms spherulites—structures of oriented crystallites and amorphous regions—through nucleation and growth [13]. Secondary crystallization, influenced by thermal history, involves further crystallization and reorganization of amorphous areas, often causing unwanted changes in polymer properties [14,15]. Crystallites (about 0.1 μm wide) have atoms arranged in a 3D lattice, while surrounding amorphous regions are disordered but may be oriented by processing. Polymer chains (1–50 μm long) span both crystalline and amorphous phases. The degree of crystallinity (θ), the weight fraction of crystalline polymer, varies from 0 to 80%. It depends on weak liquid-state bonding, molecular regularity, and strong intermolecular forces—polar groups increase crystallization tendency. Slow cooling near the crystallization temperature allows better molecular orientation and higher crystallinity, as seen in fiber or film production where chains align during drawing [16,17].

## 2. Shape Memory Polymers in Robotics

**Figure 6:**
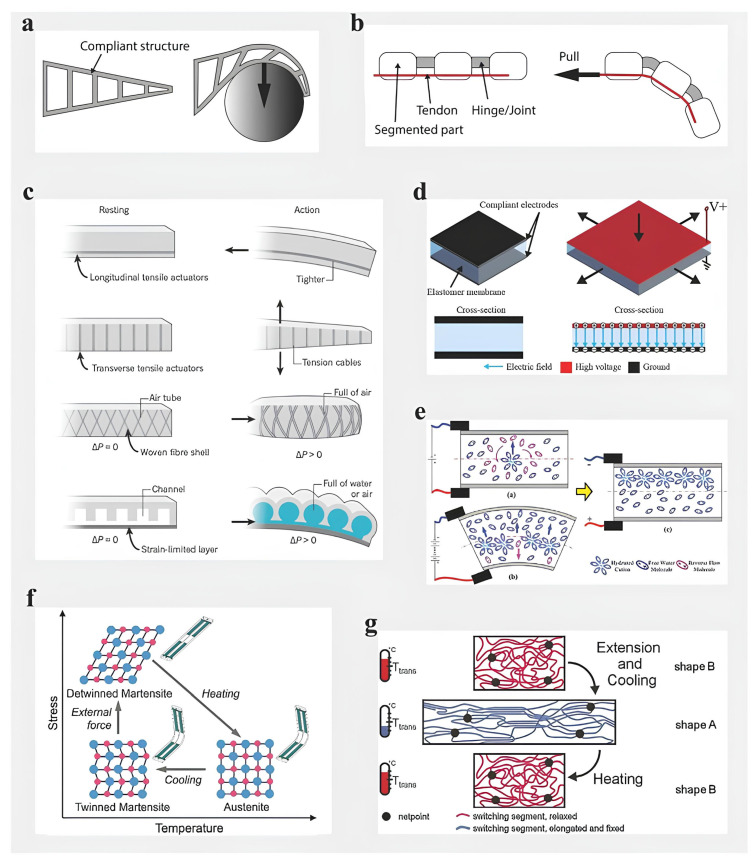
The principle of operation of technologies for soft grip. (**a**) Contact-controlled deformation (Fin Ray structure). Reproduced with permission from: Shintake, J.; Cacucciolo, V.; Floreano, D.; Shea, H. Soft Robotic Grippers. Advanced Materials 2018, 30, [24]; (**b**) Tendon-controlled actuators. Reproduced with permission from: Shintake, J.; Cacucciolo, V.; Floreano, D.; Shea, H. Soft Robotic Grippers. Advanced Materials 2018, 30, [24]; (**c**) Fluid elastomer actuators (FEA). Reproduced with permission from: Rus, D.; Tolley, M.T. Design, Fabrication and Control of Soft Robots. Nature 2015, 521(7553), 467–475 [25]. © Springer Nature; (**d**) Dielectric elastomer actuators (DEA). Reproduced with permission from: Shintake, J.; Cacucciolo, V.; Floreano, D.; Shea, H. Soft Robotic Grippers. Advanced Materials 2018, 30, [24]; (**e**) Ionic polymer-metal composites (IPMC). Reproduced with permission from: Sun, Z.; et al. A Novel Discrete Adaptive Sliding-Mode-Like Control Method for Ionic Polymer–Metal Composite Manipulators. Smart Materials and Structures 2013, 22(9), 095027 [26]. © IOP Publishing; (**f**) Shape memory alloys (SMA). Reproduced with permission from: Wang, W.; et al. Soft Composite Hinge Actuator and Application to Compliant Robotic Gripper. Composites Part B: Engineering 2016, 98, 397–405 [27]. © Elsevier; (**g**) Shape memory polymers (SMP). Reproduced with permission from: Lendlein, A.; Kelch, S. Shape-Memory Polymers. Angewandte Chemie International Edition 2002, 41(12), 2034–2057 [28]. © Wiley-VCH.

Most key components of industrial robots are fabricated from rigid materials such as metals and plastics. While the high stiffness of these materials enables robots to perform heavy-duty tasks beyond human capability, it restricts their compliance and adaptability. Grasping refers to maintaining hold of an object against external perturbations, whereas manipulation involves applying forces and moments to reposition or reorient the object relative to the manipulator frame [18]. Beyond grasping and manipulation, robotic end effectors facilitate diverse functions—including locomotion, digging, sorting, and tactile sensing—by interfacing with objects’ physical properties. However, the inherent rigidity of traditional robotic structures limits conformability to irregular targets and raises safety concerns for human interaction [19]. To mitigate these limitations, soft robotics has emerged as a paradigm shift in end-effector design, producing “soft grippers” and “soft manipulators” with enhanced adaptability and intrinsic compliance [20]. These systems utilize compliant materials, flexible actuators, and low-stiffness mechanisms, enabling safe, adaptive interactions without reliance on complex sensing or control architectures. Among actuation methods, pneumatic networks and motor-driven tendon systems currently demonstrate superior performance for integration into applications such as soft wearable devices and adaptive grippers [21]. Figure 6 provides a comparative overview of three soft gripper architectures: non-continuum bending grippers (NBG), continuum bending grippers (CBG), and continuum twisting grippers (CTG), detailing their mechanical characteristics, typical use-cases, and biological analogs [22].

Deformation of passive structures can utilize either reaction forces arising from contact with the object (Figure 6a), or it can be based on the pull of inserted cables (Figure 6b). Fluid elastomeric actuators rely on the inflation of their elastomeric chamber structure, the deformation of which is shaped using asymmetric geometry or reinforcing fibers (Figure 6c). Electroactive polymers, such as dielectric elastomer actuators (Figure 6d) and ionic polymer–metal composites (Figure 6e), actively deform in response to electrical stimuli. The shape memory effect of some materials can also be used as a means of activating a soft grip. The main materials of this type include shape memory alloys (Figure 6f) and shape memory polymers (Figure 6g). The types of soft grips described above are shown in Figure 6 [23].

### 2.1. Shape Memory Polymers

**Figure 7:**
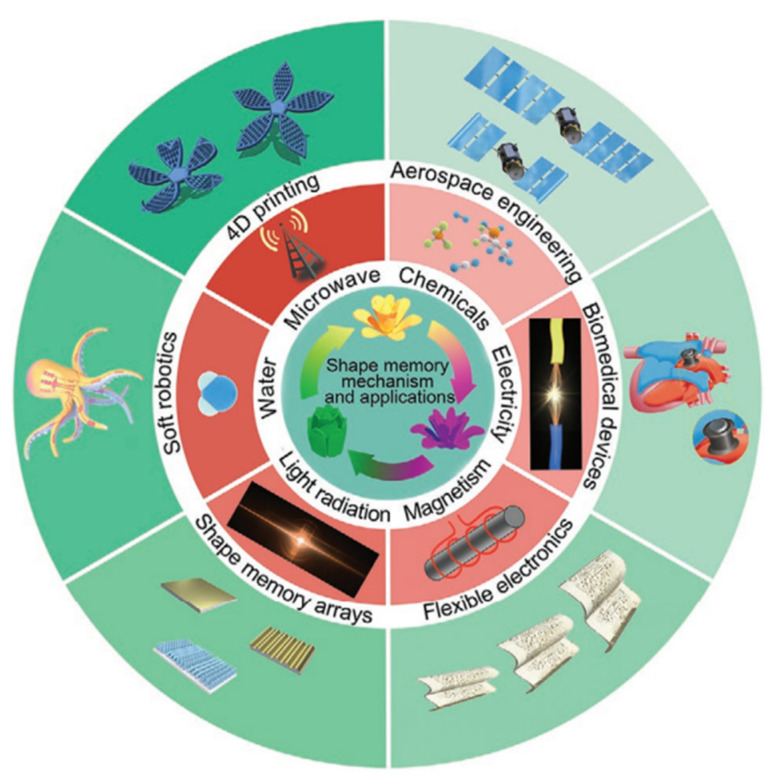
Scheme of shape memory polymers (SMP): mechanism, control methods and applications. Reproduced from *Advanced Materials*, Vol. 33, Issue 6, “A Review of Shape Memory Polymers and Composites: Mechanisms, Materials, and Applications” by Jinsong Leng, Yanju Liu, Fenghua Zhang et al., © 2020 Wiley. Reproduced with permission [29].

Shape memory polymers (SMPs), a class of stimuli-responsive materials (see Figure 7), have attracted scientific interest since the 1960s. Early studies showed that γ-irradiated polyethylene (PE) exhibits shape memory behavior across low and high-temperature cycles. SMP (shape memory polymers) are a class of materials that can transition between a temporary and a permanent shape in response to external stimuli. The permanent shape is typically stable below the phase transition temperature (T_trans_), which may vary depending on the polymer and can be above or below room temperature. Reheating above T_trans_ triggers recovery to the original shape. This effect has been demonstrated in various polymer types, including amorphous polymers, semicrystalline polymers, and liquid crystalline elastomers (LCE) [29].

**Figure 8:**
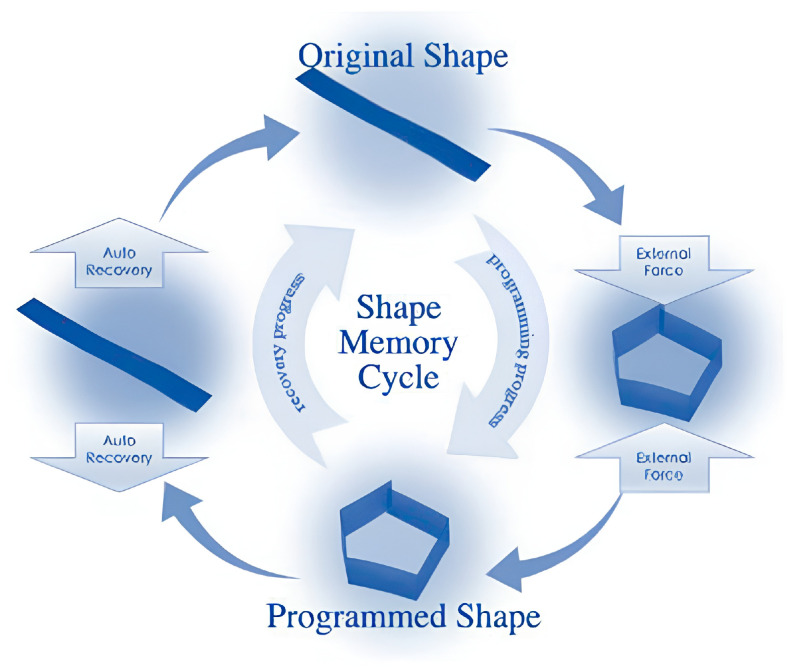
Shape memory cycle [35].

The polymer recovers its original shape upon stimulation (see Figure 8). The programming process, which defines temporary shapes, can be repeated multiple times, but the polymer has only one permanent shape. This programming is a post-synthesis treatment that determines the recovery pathway. Polymers that change shape under stimuli without programming are classified as shape-changing polymers, not SMPs. Thus, polymers subject to defined shape memory programming are correctly classified as SMPs [30].

**Figure 9:**
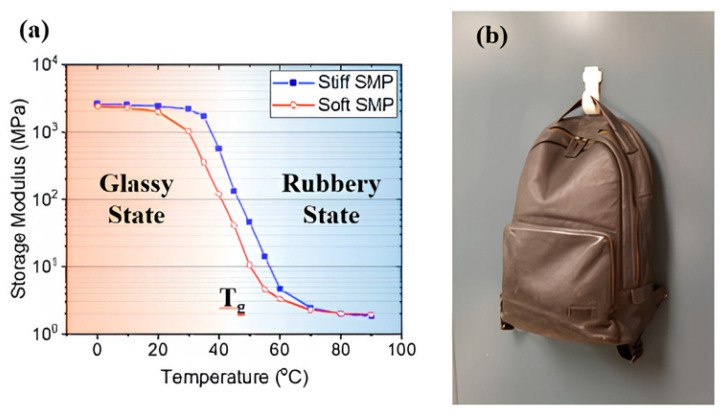
(**a**) Temperature dependence of memory modules of hard and soft shape memory polymers (SMPs); (**b**) Reversible dry adhesive hook using SMP [30].

There are two types of actively shape-changing polymers: shape memory effect (SME) polymers and shape change capability (SCC) polymers. Both use polymer networks with stimulus-responsive groups but differ in motion type and reversibility [31]. SMPs can be deformed into a temporary shape fixed until a stimulus triggers a return to the original shape, reversing the deformation [32]. SCPs gradually change shape under stimuli and revert immediately when the stimulus stops. SMPs and shape memory alloys (SMAs) are used in grippers due to their shape recovery and stiffness changes. SMPs are more flexible, less stiff, and cheaper than SMAs but research mostly focuses on gripping rather than grasping [33]. Some SMP grippers respond to stimuli like light or moisture and use friction or suction to grip objects, similar to grain-jamming grippers. SMPs soften when heated, forming tight contact with surfaces under pressure, and solidify when cooled, maintaining adhesion. This adhesion is reversible with reheating. Figure 9 shows SMP storage moduli and their use as adhesive hooks [34].

### 2.2. Molecular Mechanism of Shape-Memory Effect

**Figure 10:**
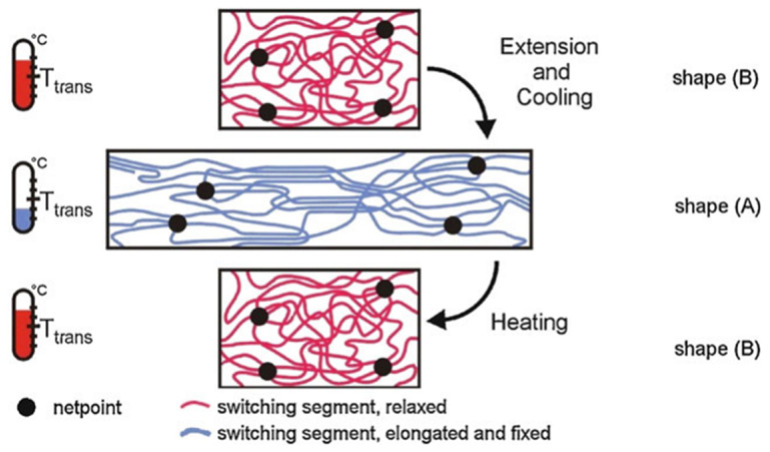
Molecular mechanism of thermally induced SME. T_trans_ is the thermal transition temperature of the switching phase [38].

While the previous section outlined the practical behavior of SMPs in soft grippers, this section delves into the molecular mechanism behind the shape memory effect. The shape memory effect (SME) requires a polymer network architecture combined with tailored processing, termed the Shape Memory Creation Process (SMCP). The polymer network consists of permanent network points and stimulus-sensitive molecular switches. Permanent shape is fixed by network points—either covalent or physical cross-links—connected by flexible chain segments that enable elastic deformability and orientation [36]. Temporary shape fixation occurs by reversible stiffening of switch domains or formation of reversible cross-links, stabilizing the deformed conformation. In multiphase polymers, hard domains with the highest thermal transition temperature (T_perm_) serve as physical network points, while switch domains at a lower transition temperature (T_trans_) act as molecular switches. These switches form reversible bonds to fix and release temporary shapes upon stimulus (see Figure 10) [37].

**Figure 11:**
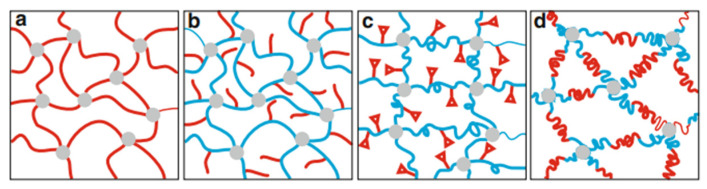
Examples of polymer network architectures suitable for SME exposure (molecular switches—red; network points—gray). (**a**) Switch segments connecting network points; (**b**) Side chains as switch segments; (**c**) Functional groups as molecular switches capable of reversibly forming a covalent binding; (**d**) ABA triblock segments connecting network points. Reproduced with permission from Behl et al., in Shape-Memory Polymers, Springer, 2009 [40].

Physical cross-linking occurs during cooling via solidification of switch domains, such as vitrification or crystallization, which reverses upon reheating. Chemical cross-linking forms via reactions between functional groups—e.g., cinnamic acid derivatives—that create reversible bonds cleavable on demand. SMPs are classified by their molecular switching mechanisms [36,39]. Figure 11 illustrates common SMP network architectures:

(a) Covalently cross-linked networks with switch segments that fix temporary shapes via solidification at T_trans_ (often Tg). If switch segments crystallize, the temporary form is semicrystalline.
(b) Networks with dangling side chains for added flexibility.
(c) Networks with reversible covalent bonds controlled by external stimuli.
(d) ABA triblock copolymer networks forming multiphase morphologies; miscible or phase-separated blocks can act as switch domains [40].

### 2.3. Types of Shape Memory Polymers

Shape memory polymers (SMPs) represent a broad and versatile class of smart materials that can be categorized based on their molecular structure, type of crosslinking, and activation mechanisms. These differences determine key functional characteristics such as mechanical strength, shape recovery behavior, reprogrammability, and suitability for specific application environments. This subsection focuses on three representative types of SMPs that are particularly relevant in the context of soft robotics. Thermoplastic SMPs are physically crosslinked materials that allow for reversible programming and reshaping due to their heat-dependent softening and hardening behavior. Covalently crosslinked SMPs, on the other hand, form chemically stable networks that offer higher thermal and mechanical stability, making them suitable for applications requiring durability and structural integrity. A third category, magnetic shape memory polymers, combines polymer matrices with magnetic fillers, enabling remote, contactless actuation through external magnetic fields. Each of these types offers specific advantages and faces unique limitations. In the following sections, we discuss their properties in more detail, with emphasis on their mechanisms of action and potential use in the design of soft robotic grippers and actuators.

#### 2.3.1. Thermoplastic Polymers

Shape memory thermoplastic polymers (SMPs), particularly thermoplastic polyurethanes (TPUs), are an important group of materials that have gained the attention of researchers due to their reversible deformation capability, ease of processability, and sensitivity to temperature changes. Their structure is based on physical cross-linking through phase-separated domains—soft segments that respond to stimuli and hard regions that provide mechanical support. This system allows the realization of shape memory without the need for permanent chemical bonds [40,41].

One of the most studied systems are poly(ε-caprolactone) (PCL)-based poly(ε-caprolactone) (PCL) polyurethanes, where the PCL segments function as thermally activatable switching units (T_m_ ≈ 44–45 °C) and the oligourethane or aromatic segments form rigid domains that provide stability and flexibility to the material [42]. TPUs based on this base exhibit a high degree of shape fixation and recovery, making them suitable material for applications in soft robotics and biomedical fields.

Currently, research focuses on modifying the properties of TPUs by optimizing the composition of copolymers, the ratio between soft and hard segments or different processing techniques. Introducing modifications in the crystalline or amorphous phase and combining block copolymers has allowed us to achieve programmable deformation and adjustable transition temperature [43]. Moreover, rapid developments in 4D printing allow precise control of the actuation behavior in individual parts of the TPU material, leading to the fabrication of complex moving structures such as tentacles and actuators with localized shape changes [44].

The addition of functional nanoparticles such as carbon nanotubes, Fe_3_O_4_ or cellulose nanocrystals significantly enhances the activation sensitivity, mechanical strength and thermal conductivity of TPUs. The nanocomposite thermoplastic SMPs thus formed are promising for applications that require a fast and repeatable response to external stimuli—such as in wearable electronics, soft prosthetics, or sensing [45].

Despite the advantages in processability and repeatability, TPUs may exhibit limited stability at higher temperatures compared to permanently cross-linked polymers. For this reason, increased research is focusing on hybrid materials that combine a thermoplastic matrix with dynamic linkages or double networks to improve the balance between structural integrity and reprogrammability.

Overall, thermoplastic SMPs—especially TPUs—can be considered as key materials in the field of smart and adaptive systems. Their favorable processability, biocompatibility and wide range of applications make them a promising platform for the development of innovative robotic solutions, medical devices and functional moving parts.

Linear block copolymers are key physically cross-linked SMPs. Notable examples with shape memory characteristics (SMCs) and melting points (T_m) as transition temperatures (T_trans) include polyurethanes and polyetheresters. In polyesterurethanes, polyester segments like poly(ε-caprolactone) (PCL, T_m = 44–45 °C) act as switching domains, while oligourethane segments form hard domains.

**Figure 12:**
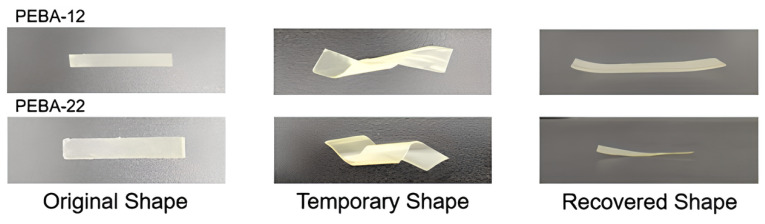
Photos of shape memory behavior for PEBA-12 and PEBA-22 [47].

Figure 12 illustrates thermoplastic SMPs composed of binary mixtures of multiblock copolymers, where one component forms hard domains and the other switching domains [46].

#### 2.3.2. Covalently Crosslinked Polymers

**Figure 13:**
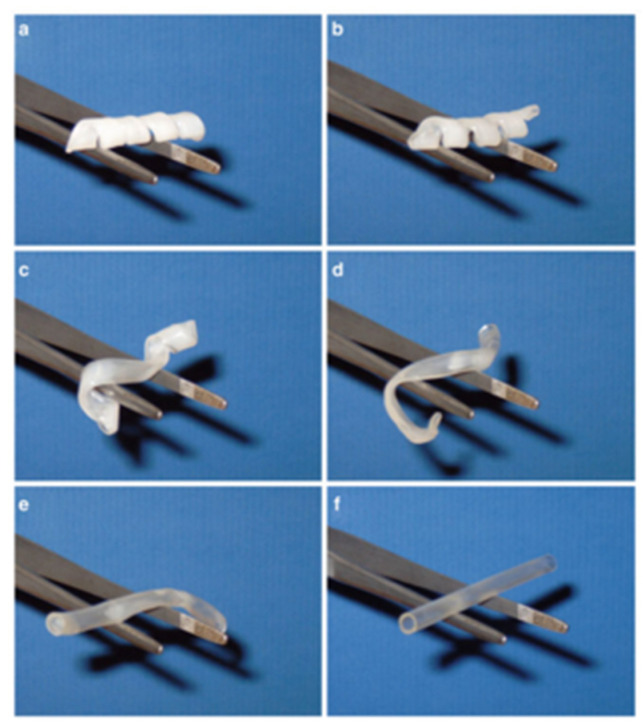
Series of photographs showing recovery of a shape-memory tube: (**a**–**f**) start to end of the recovery process; total time, 10 s at 50 °C. The tube was prepared from poly(εcaprolactone)dimethacrylate (Mn104) and had been deformed and fixed in a corkscrew-like temporary shape Reproduced from: Lendlein, A. and Kelch, S. (eds.), Shape-Memory Polymers, *Springer*, **2010**, with permission of the publisher [48].

Covalently crosslinked shape memory polymer (SMP) networks are formed via crosslinking of polymers or (co)polymerization involving trifunctional monomers. Crosslinking occurs during synthesis or post-processing, such as radical initiators or radiation. Semicrystalline polycyclooctene, synthesized by ring-opening metathesis and crosslinked with dicumyl peroxide, exhibits a shape memory transition (T_trans_) equal to its melting temperature (T_m_), tunable by trans-vinylene content. Higher crosslink density reduces crystallinity. It recovers shape rapidly (0.7 s at 70 °C) and shows strain-dependent anisotropy during cooling, confirmed by WAXD. Mechanochromic dyes track T_trans_ by changes in absorption. Another method uses copolymerization of monofunctional monomers with bifunctional crosslinkers like oligo(ε-caprolactone)-dimethacrylates (see, producing AB-type SMPs with adjustable T_trans_ = T_m_. Soft poly (n-butyl acrylate) domains provide elasticity near application temperatures. Photopolymerized networks with polyhedral oligosilsesquioxane (POSS) moieties show T_trans_ from oligo(ε-caprolactone) melting, and at 47% POSS content, a second rubbery plateau emerges from POSS interactions (see Figure 13) [48,49].

#### 2.3.3. Magnetic Shape Memory Polymers

**Figure 14:**
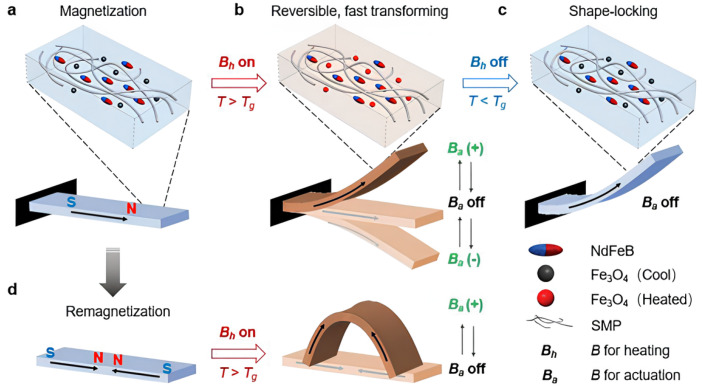
Mechanism of operation of magnetic shape memory polymers (M-SMP): (**a**) M-SMP is solid at low temperatures and cannot be activated by an applied activation field (B_a_); (**b**) By applying a heating magnetic field (B_h_), the M-SMP softens and can be activated; (**c**) After switching off B_h_, the M-SMP cools down and solidifies, locking the activated shape; (**d**) The magnetization profile of the M-SMP can be reprogrammed to allow for a different activation Reproduced from Ze et al., Advanced Materials, 2020, 32(4), 1906657 [49]. © 2020 Wiley. Reproduced with permission.

Shape-programmable soft materials enabling multifunctional shape manipulation—such as reprogrammable, unbound, rapid, reversible transformations, and shape locking—under external stimuli (heat, light, magnetic fields) are essential for soft robotics, actuators, portable, and biomedical devices. The M-SMP system incorporates Fe_3_O_4_ and NdFeB magnetic particles within an amorphous SMP matrix [49]. Fe_3_O_4_ particles facilitate induction heating under high-frequency alternating magnetic fields, enabling shape locking and unlocking. NdFeB particles, magnetized with designed profiles, enable programmable deformation under applied magnetic fields (see Figure 14) [50].

Figure 14 illustrates the M-SMP working mechanism. M-SMP containing NdFeB and Fe_3_O_4_ particles is magnetized with a targeted profile (e.g., longitudinally) using a pulsed magnetic field (~1.5 T). At room temperature, the material is rigid and resists deformation by the pulsed field (Ba). Applying a high-frequency alternating magnetic field (B_h_) induces heating of Fe_3_O_4_ particles, raising the M-SMP above its Tg and significantly reducing its modulus, allowing easy bending with a small Ba. Alternating Ba’s direction enables rapid up/down bending. Removing B_h_ cools and stiffens the M-SMP, locking the shape without Ba. The magnetization can be reprogrammed via remagnetization, enabling reconfigurable shape changes—for example, folding activation by remagnetizing the beam while mechanically locked in a folded state [49].

## 3. Polymers in Soft Robotics and New Trends

In recent years, polymers have become a foundational material in the development of soft robotics—an emerging field inspired by the flexibility and adaptability of biological systems. Soft robotic systems, particularly grippers, leverage the unique characteristics of polymers—such as their elasticity, shape-morphing capabilities, and responsiveness to external stimuli—to create functional and highly adaptable devices. This chapter highlights three major research directions in the domain of polymer-based soft grippers, each representing significant technological and application-oriented advancements.

Additively manufactured soft grippers, produced through advanced 3D printing techniques, facilitate the rapid and precise fabrication of complex geometries using soft polymeric materials. These methods offer a high degree of design flexibility, reduce production costs, and allow for the seamless integration of functional materials during the printing process.

Hydrogel-based soft grippers represent another innovative class, capitalizing on the hydrogels’ capacity to absorb water and undergo significant changes in volume or mechanical properties in response to environmental stimuli. Due to their high biocompatibility and sensitivity to physical and chemical changes, these grippers are especially promising for biomedical and microfluidic applications.

These emerging trends underscore the rapid progress in soft robotics, with polymer-based materials playing a central role in expanding the functional scope, adaptability, and real-world integration of next-generation robotic systems.

An important contribution to the development of smart materials for soft robotics is a review paper by Scalet [50], which discusses shape-memory polymers with repeatable bidirectional and multidirectional effects, and their potential for actuators and adaptive grippers. This review study provides a comprehensive look at two-way and multiple-way shape memory polymers (SMPs) that are capable of repeatedly changing shape without the need for a complex external mechanical system. The author describes the principles of the two-way shape effect, material requirements, crosslinking types, and activation methods. These materials are particularly relevant to the field of soft robotics, as they enable the creation of actuators and grippers that can move, adapt shape, and respond repeatedly to an external stimulus (e.g., temperature), without conventional motors or hydraulics. Their flexibility and programmability make them a suitable basis for biomimetic systems and wearable robotic elements that need to be lightweight, safe and adaptable. Below are further examples of the use of polymers in soft robotics.

### 3.1. Grippers Based on Shape Memory Polymers

Grippers based on Shape Memory Polymers (SMPs) represent one of the most promising technologies within the domain of soft robotics. Among these materials, shape memory polymers (SMPs) are particularly promising due to their ability to undergo controlled deformation and recovery, making them suitable for use in soft grippers. A more detailed discussion of their properties and mechanisms follows in Section 2.1. This unique functionality enables their use as active components that can autonomously undergo shape transformation without the need for complex mechanical or electronic actuators. In the context of soft robotic grippers, SMPs facilitate the development of self-adjusting and reusable end-effectors capable of delicately manipulating objects of varying shapes and sizes. Their inherent material simplicity, low weight, and ability to undergo programmable deformation make them an attractive solution for a wide range of applications, including medical devices, precision assembly, automated handling systems, and exploration in confined or hazardous environments.

This chapter explores the fundamental operating principles of SMPs, their material characteristics, activation mechanisms, and design strategies relevant to gripper integration. Additionally, it addresses current technical challenges and highlights emerging trends and research directions in the development and application of SMP-based soft grippers.

#### 3.1.1. Magnetic Shape Memory Gripper

**Figure 15:**
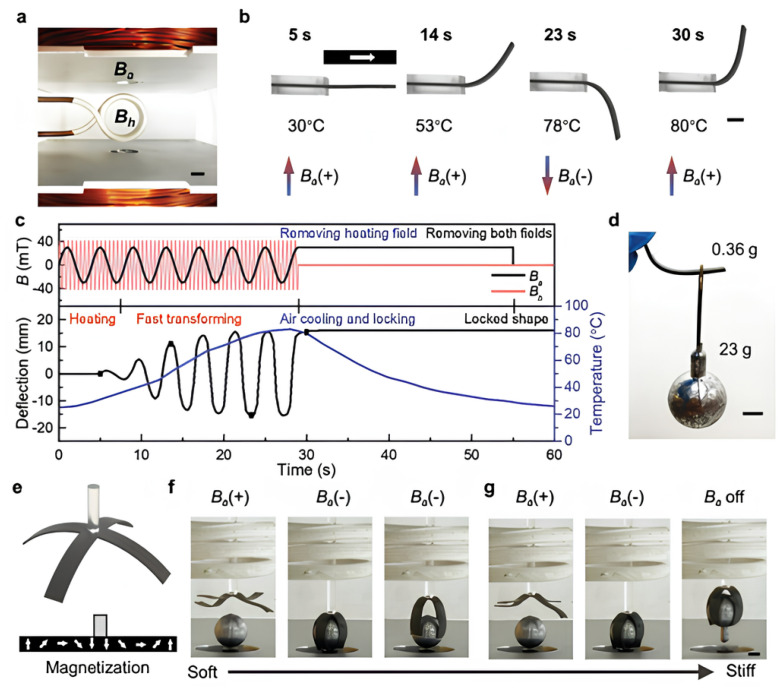
Fast-transforming and shape locking of M-SMPs via superimposed magnetic fields. (**a**) Experimental setup for the superimposed magnetic fields: a pair of electromagnetic coils generates the actuation magnetic field, B_a_; the solenoid in the middle generates the heating magnetic field, B_h_. (**b**) Cantilever bending and shape locking. (**c**) Magnetic field profiles of B_a_ and B_h_ and beam deflection and temperature with respect to time. (**d**) Locked bending beam carrying a weight (23 g) that is 64 times heavier than its weight (0.36 g). (**e**) Design and magnetization profile of a four-arm M-SMP gripper (0.47 g). (**f**,**g**) M-SMP gripper lifting a lead ball (23 g) without (**f**) and with (**g**) shape locking. Scale bars: (**a**): 15 mm; (**b**,**d**,**g**): 5 mm. Reproduced from Ze et al., Advanced Materials, 2020, 32(4), 1906657 [49]. © 2020 Wiley. Reproduced with permission [49].

In Figure 15e, the construction and directions of magnetization of the four-arm gripper are shown. By the action of B_h_ and positive Ba (upward), the tentacle softens and opens for grasping. By switching Ba to a negative value, the gripper adapts to the lead ball. At this moment, the ball will slip out if the gripper is raised (see Figure 15f). However, as shown in Figure 15g, the tentacle can solidify and fix in the activated shape after we remove B_h_. It can then effectively lift the lead ball without any external stimulation [49].

In addition, the fast and reversible activation of the M-SMP-based gripper enables multiple uses and reconfigurable gripping motions, which is advantageous compared to conventional SMP-based grippers that allow only a single gripping operation.

#### 3.1.2. Adhesive SMP Gripper

Soft robotic tentacles are the subject of extensive research due to their ability to adapt their morphology to grasp objects. In general, it is highly desirable for a gripper to have unbounded, reversible actuation with the ability to lift or hold heavy objects without continuous energy input. A shape memory gripper is a simple soft robot that can perform grasping and lifting activities. It only requires programmed bending and recovery behavior from polymers, making it easy to assemble using a variety of SMPs with desired mechanical properties [51].

**Figure 16:**
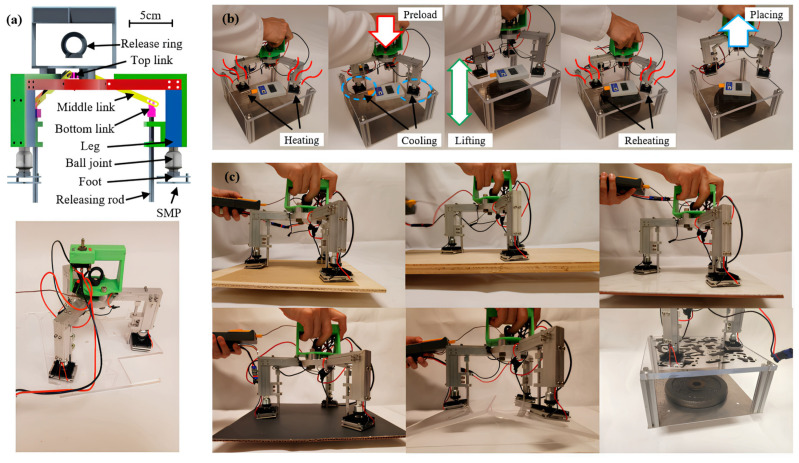
(**a**) Computer-aided design (CAD) and photo-adhesive SMP gripper. The gripper includes a battery and thermocouples for heating/cooling and temperature sensing; (**b**) An SMP adhesive gripper is used to demonstrate pick-and-place functionality; (**c**) The images show how the SMP adhesive gripper grips sandpaper, wood board, tile, poster paper, and angled acrylic board, as well as acrylic board wetted with blue-stained water [30].

A computer-aided design (CAD) and photograph of the SMP adhesive gripper, including three legs and three feet, is shown in Figure 16 Ball joints with a maximum rotation angle of 35° connect the legs and heels. The upper element and the lower element are connected to the middle elements using joints with pins in the grooves. Most of the body is made from machined aluminum and 3D-printed parts. All three legs are connected in parallel to a lithium-polymer (LiPo) battery with a voltage of 11.1 V and a capacity of 1200 mAh (Kinexsis) [52]. The wires between the legs and the outer guide of the gripper are hidden in aluminum frames. The three TECs are used for the heating and cooling cycles and are located under the three legs [52]. To control the TEC, a 15A maximum double-pole, double-throw (DPDT) switch with a “center off” position is used, which switches the electrical polarity and thus the direction of current flow.

**Figure 17:**
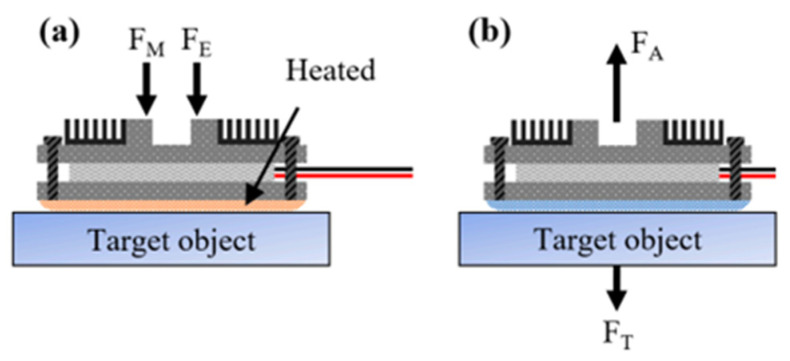
Free-body diagram of the heel of the SMP adhesive gripper: (**a**) Forces during preload; (**b**) Forces during picking [30].

In Figure 17b, the overall pick-and-place step is shown, in which the SMP is first heated to more than 90 °C, pre-loaded, and then simultaneously cooled to 40 °C. Then the SMP gripper lifts the stage with an added 4.5 kg weight. Finally, the SMP is reheated to above 90 °C, and a release force is applied to place the weight [53]. The SMP is heated and brought closer to the target object in the pick-up step, and the ball joints between the legs and feet are passively rotated to achieve uniform contact. The step of applying the preload, which is the sum of the gripper mass (F_M_) and the external force (F_E_), is shown in Figure 17a. Since the preload is very important for adhesion performance, it is desirable that F_E_ is high.

### 3.2. Additively Produced Soft Grippers

Additive manufacturing, particularly 3D printing, is introducing significant advancements in the development of soft robotic grippers. It enables the rapid and precise fabrication of complex geometries using soft polymeric materials, while offering a high degree of design flexibility and the potential for integrating multiple functionalities directly during the printing process. In addition to conventional 3D printing, this chapter also addresses the emerging concept of 4D printing, which involves the use of smart materials capable of changing their shape or properties over time in response to external stimuli. This innovative approach paves the way for the development of active and adaptive grippers with enhanced functionality.

The chapter focuses on the materials, manufacturing techniques, advantages, limitations, and current trends related to additively manufactured soft grippers, including future perspectives on the application of 4D printing in soft robotics.

A practical demonstration of the use of shape memory polymers in the field of soft robotics is presented by Yang et al. [54], where the authors developed a 3D printed thermally activated polyester-urethane-based gripper with repeatable actuator behavior. In this research, a thermally active SMP based on polyester-urethane (PEU), processed by 3D printing (FFF technology), was designed and tested. The result was a functional soft robotic gripper that was able to repeatedly perform precise grasping and manipulation of objects. After programming and subsequent heat activation (~60 °C), the material achieved a reversible deformation of approximately 16% over more than 100 cycles, demonstrating high repeatability and reliability. In experiments, the gripper was able to safely lift and carry fragile objects such as an egg, confirming its practical applicability in soft robotics for tasks where delicate and adaptive movements are required—for example, in healthcare, food or micro-manipulation. The study thus provides important evidence that SMP materials can fully replace traditional robotic actuators in soft systems. Below are other examples of soft grippers made by additive manufacturing [51].

#### 3.2.1. Soft Grasping Mechanisms with Inward Deformation

**Figure 18:**
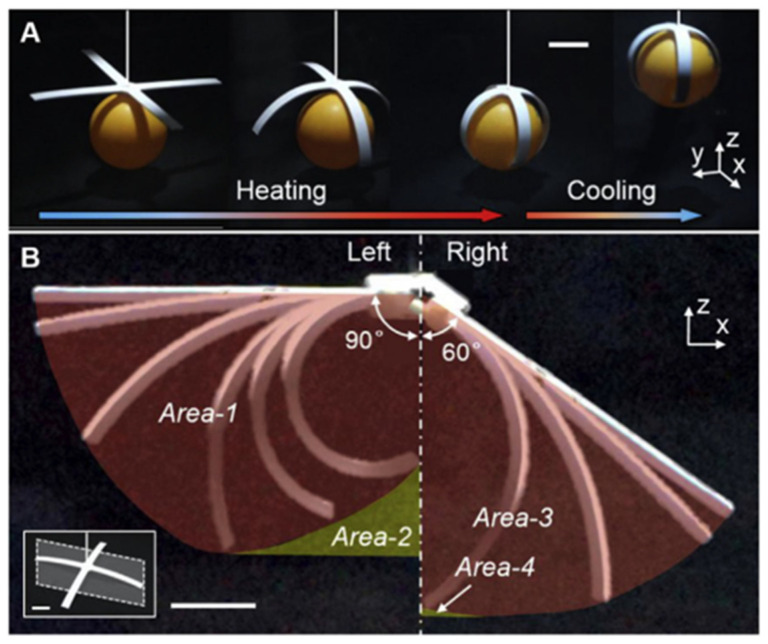
A direct-printed flat gripper is used to grip a 40 mm diameter ping-pong ball (**A**) and a comparison of the accessible and inaccessible areas of individual arms of grippers with two different architectures (**B**). Scales: 10 mm. Reprinted from Composites Part B: Engineering, Vol. 164, Wei Wang, Chak Yuk Yu, Pablo Antonio Abrego Serrano, Sung-Hoon Ahn, Soft grasping mechanisms composed of shape memory polymer-based self-bending units, pp. 198–204, Copyright (2019), with permission from Elsevier [58].

Each arm of the gripper can be considered a self-propelled specimen with dimensions of 50 mm in length, 4 mm in width, and 1.1 mm in thickness, with all printed PE fibers parallel to the length direction [55]. A flat gripper was used to grip a 40 mm diameter ping-pong ball, as shown in Figure 18. The structures were activated in an oven with an internal temperature set at 90 °C. The stiffness of each branch was switched from a high state to a low state during the heating process, causing each branch to produce in-plane bending deformation simultaneously, with all four branches used to grip the ball [56]. During the subsequent cooling process, the bending strain of each branch was maintained in a high stiffness state to hold the ball at an ambient temperature lower than the T_g_ of PE [57]. During the gripping process, the in-plane area (i.e., accessible area) of each branch surrounded by the tip trajectory of the bent sample is shown in red (area-1), while the inaccessible area between the tip trajectory and the ground is shown in yellow (area-2), as described in Figure 18B [58].

#### 3.2.2. Three-Dimensional Printed Hydrogel Soft Gripper

Another potential application of hydrogels in soft robotics is the development of 3D-printed soft actuators utilizing polyelectrolyte hydrogels for electromechanical activation. This study introduces the design, fabrication, and performance assessment of such an actuator, focusing on the exploitation of ionic conductivity and swelling behavior under electrical stimulation to achieve controlled deformation. These characteristics make the material highly suitable for integration into soft robotic systems [59].

**Figure 19:**
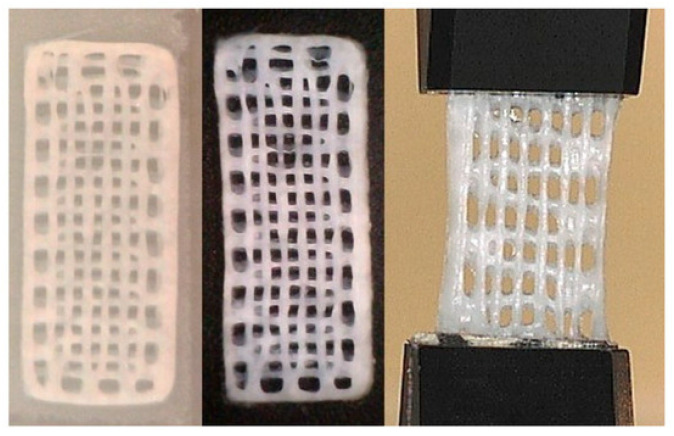
Printed actuator samples [60].

The actuators (see Figure 19) were manufactured using advanced 3D printing techniques, ensuring precise geometrical configurations and reproducible actuation performance. A cationic hydrogel network was engineered to respond to applied electric fields through asymmetric water migration, producing bending motion independently of external aqueous environments. This functionality is essential for practical applications operating under ambient and dry conditions.

Another potential application of hydrogels in soft robotics is the development of actuators that leverage internal ion-induced water migration for motion generation, rather than relying on external osmotic gradients. Among the key findings of this study:

- The actuator’s bending behavior results from internal ionic water transport, eliminating the need for environmental hydration gradients.
- Incorporation of graphene enhances thermal dissipation, reducing water evaporation and thereby extending actuator lifespan.
- Hybrid pNIPAM/pAAM hydrogels exhibited a sweating effect—autonomous water release—that opens new avenues for biomimetic system design.
- Strategies to improve air stability include encapsulation techniques and hybrid organohydrogel architecture.

This work represents a significant step forward in soft actuation technologies, combining material chemistry, 3D printing, and electroactive hydrogel performance to address operational challenges such as water retention and mechanical durability in ambient conditions.

#### 3.2.3. Four-Dimensional Printing in Soft Robotic Grippers Creation

Four-dimensional printing builds upon 3D printing by adding a time-dependent element—printed objects are no longer static but capable of autonomously changing, transforming, or activating over time in reaction to environmental triggers such as temperature, moisture, light, or electrical fields. Put differently, the “fourth dimension” in 4D printing refers to the capacity of the object to modify its shape or characteristics after manufacturing. This enables the creation of dynamic, self-regulating systems like soft robotics, self-folding structures, or intelligent materials that interact with their environment [61].

**Figure 20:**
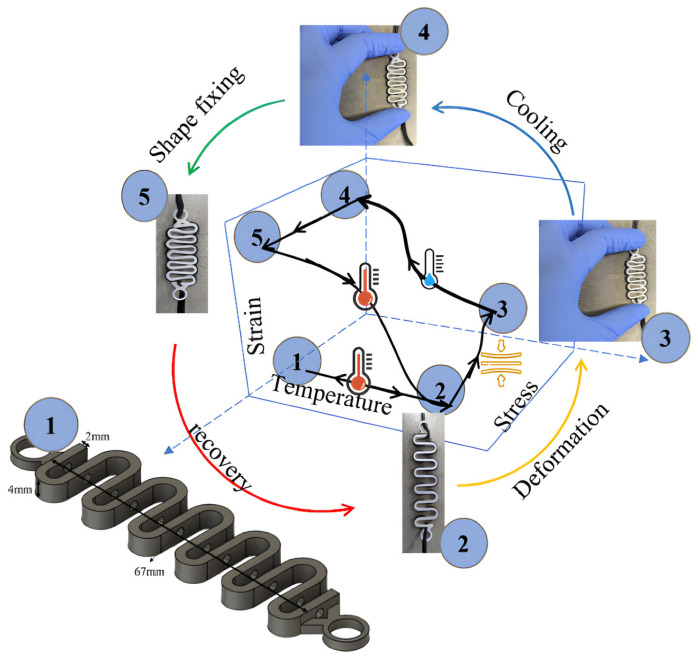
The principle of 4D printing technology [64].

In essence, 4D printing integrates the geometric precision of 3D printing with smart materials that can adapt and respond actively to external conditions. One of the possible fabrication methods is additive manufacturing. This type of technology enables the integration of time-dependent deformation directly into 4D-printed structures, effectively transforming them into autonomous shape-changing systems. The article titled “Pattern-driven 4D Printing” [62,63] introduces a novel design strategy based on spatial patterning, where the controlled deposition of materials defines the mechanical and activation behavior of printed objects when exposed to thermal stimuli (see Figure 20) [64].

The core innovation lies in exploiting the differential thermal expansion between a passive material (PLA) and an active, thermally responsive material (TPU), both printed in distinct, tunable patterns. By adjusting print angles, layer infill, and material distribution, the research team was able to pre-program complex, predetermined deformations into flat 2D prints that transform into 4D configurations upon heating. This methodology eliminates the need for complex multi-material assemblies or embedded electronics, offering a low-cost and scalable pathway to 4D-printed soft robotic components.

A computational model was developed to predict bending behavior based on geometric and material parameters. This model was experimentally validated and demonstrated strong agreement with the observed deformations. The authors further showcased the versatility of this technique by fabricating various structures, including foldable boxes, flower-shaped actuators, and a thermally triggered gripper, illustrating the potential of this method for autonomous control and self-deploying robotics.

From a technical standpoint, this work represents a significant contribution to the fields of soft robotics and smart materials. It introduces a “design-to-function” paradigm, in which printed material patterns directly dictate mechanical actuation. This approach not only reduces system complexity but also enhances the potential for miniaturization and integration in robotic and biomedical applications [65].

Four-dimensional printing is used in soft robotics because it enables the integration of functionality directly into the fabrication process. Unlike traditional manufacturing, where sensors and actuators are added after construction, 4D printing embeds functional materials at precise locations during printing. This allows the robot to change shape or behavior in response to external stimuli, such as heat, light, or moisture. According to the article “Closed-loop 4D-printed soft robots” [66], the functionality of a soft robot is introduced during the printing process. To control this functionality, specific functional materials are embedded in desired locations.

This method supports the development of closed-loop systems, where soft robots can autonomously sense, decide, and act without external control. By combining 4D printing with machine learning, researchers can design stand-alone robots capable of adaptive behavior in real-time. These systems promise major advancements in fields like biomedical devices, wearable technologies, and environmental sensing. Ultimately, 4D printing transforms soft robotics from passive structures into intelligent systems, enabling a new generation of responsive, autonomous machines.

#### 3.2.4. Fused Filament Fabrication in Soft Robotics

In a study by Chalissery et al. [67], the possibility of using fused filament fabrication (FFF) technology for the direct additive manufacturing of shape memory polymer (SMP) actuators, which are capable of autonomous motion in response to thermal stimuli, was investigated. The research focused on polyurethane SMPs that were printed as functional 3D structures with well-defined geometric properties, with the material itself incorporating built-in thermal activation capability.

After printing, the samples were programmed into a temporary shape by cyclic mechanical reshaping above the transition temperature (T_trans_), then cooled to fix the temporary shape. Upon re-warming above T_trans_, the objects returned to their original (permanent) shape. The authors demonstrated that the extruded actuators are able to perform repeatable motion over several cycles without significant loss of functionality, which is crucial for practical applications in robotic systems.

An important result of the work is the demonstration that functional motion and shape changes can be programmed during the fabrication of the geometry itself, eliminating the need for an additional mechanical or electronic system to control the motion. Thus, the authors show the significant potential for integrating SMP actuators directly into robotic components, such as soft grippers, where low weight, flexibility and the ability to adapt to different object shapes are required.

Another contribution of this study is to evaluate the advantages of FFF as a manufacturing technology that enables the rapid, affordable and precisely controlled creation of shape memory components from thermoplastic SMPs. The results show that actuators fabricated in this way have good cyclic stability, short response time and the possibility of localized deformation programming, which predisposes them to applications in areas such as soft robotics, biomedical devices, wearable technology or adaptive manipulators in automated systems. The study by Chalissery et al. thus provides important evidence that additive manufacturing using FFF is an effective route for the development of integrated SMP actuators, while also creating scope for further research in the combination of shape memory, custom printing and robotic functionality.

### 3.3. Hydrogel-Based Soft Grippers

Hydrogel-based soft actuators enable soft robots to execute a range of functions by modulating the deformation of morphable materials. For instance, such actuators facilitate tasks like grasping fragile items, as well as performing various forms of locomotion, including swimming, walking, crawling, and even jumping. To comprehend how hydrogels are utilized in these systems, it is important to first explore the different modes of soft actuation. The movement generated by soft actuators stems from shape deformations initiated by specific actuation methods. These methods may rely on the material’s responsiveness to external stimuli—such as electric or magnetic fields, light, or temperature—or may employ fluidic or tendon-driven mechanisms. Resulting deformations can manifest expansion, contraction, bending, twisting, or more intricate folding behaviors.

**Figure 21:**
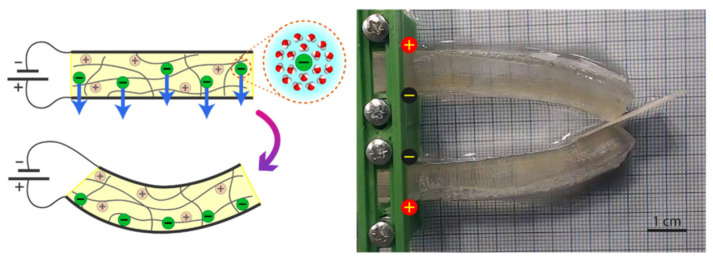
Electromechanical response of cationic hydrogel actuator under ambient conditions [68].

To address the limitations in strength and often speed associated with hydrogel-based smart actuators, alternative energy sources—such as pressurized air—have recently been explored. Fluidic actuators, particularly pneumatic types, are commonly used in soft grippers and manipulators and are typically fabricated from inert elastomers, as actuation is driven by pressurized fluid rather than by the material itself. Hydrogel-based fluidic actuators operate outside of aqueous environments to prevent undesired swelling, which is a viable strategy since water does not serve as the actuation medium in this configuration [63]. The use of hydrogels in these actuators offers additional functional advantages beyond basic mechanical motion (see Figure 21). For instance, Mishra et al. developed a conventional pneumatic actuator that exhibits autonomous sweating, enabled by the chemo-mechanical behavior of two hydrogels—poly(N-isopropylacrylamide) (pNIPAM) and polyacrylamide (pAAM)—which dynamically open and close pores in response to temperature changes. This feature contributes to extended operational lifespan. Furthermore, the incorporation of iron oxide and silica nanoparticles into the hydrogel matrix enhances the mechanical robustness of the actuator.

In Figure 21 an electromechanical response of cationic hydrogel is shown. Free chloride anions migrate toward the positive electrode, dragging free water within the hydrogel and creating a swelling gradient that induces bending of the hydrogel rod toward the negative electrode. This mechanism enables the fabrication of soft grippers composed of two parallel hydrogel rods actuated by opposing electric fields (in this setup, the negative pole is positioned internally due to the nature of the hydrogel). Electrodes are implemented as simple aluminum foil strips attached to the hydrogel. Additionally, the concept extends to complex morphing structures, such as (a) a foldable cube operating in aqueous conditions based on thermoresponsive pNIPAM/LDPE bilayer actuators and (b) a compact, air-operable actuator based on pNIPAM, encapsulated for operation in dry environments.

Another critical challenge is controlling the swelling behavior of hydrogel materials. In aqueous environments, hydrogels with a high density of hydrophilic functional groups and large pores may swell to such an extent that their mechanical properties or conductivity degrade, ultimately compromising performance. However, this issue can be readily addressed from a chemical standpoint by using a mixture of hydrophobic and hydrophilic monomers, increasing the crosslinking density, or synthesizing interpenetrating double networks. Nevertheless, most robotic applications require operation outside of water, as this aligns with our living environment. Therefore, to expand the practical applicability of these materials, it is essential to eliminate their dependence on external aqueous environments. One strategy for enabling hydrogel-based bending actuation in air can be the utilization of the water inherently retained within the hydrogel structure. With this objective in mind, we have recently developed an electroactive hydrogel based on a cationic polymer network, capable of undergoing bending deformation outside of aqueous media. Unlike previously described systems that rely on an osmotic pressure gradient from the external environment, this hydrogel leverages its intrinsic ionic conductivity and high-water absorption capacity to draw internal water toward one side [68]. This results in localized swelling on that side and contraction on the opposite side, inducing bending behavior that can be harnessed in robotic grippers.

However, this approach introduces a new challenge: maintaining constant internal water content by preventing evaporation. To address this issue, graphene was incorporated into the hydrogel to enhance its thermal dissipation, thereby reducing evaporation. This plays a crucial role in extending the operational lifespan of the hydrogel-based actuator.

Additional strategies to mitigate water loss include encapsulating the hydrogel in a thin, flexible, and bendable coating, developing hybrid organohydrogels, or incorporating inorganic salts and nanoparticles. Nonetheless, like underwater actuators, the response time of this system remains relatively slow [66,69].

## 4. State of the Art Analysis

To express the relevance of the topic of this study, two datasets with approximately equal numbers of published materials were analyzed. The selected datasets are Web of Science and Scopus. These datasets are multidisciplinary and fall under the open access category. The search for publications of various types was conducted using the keywords relevant to this issue: soft, gripper, polymer. Publications were analyzed according to three criteria: publication year (2015–2024), publication category according to keywords, and type of published document. Subsequently, graphs were generated to visualize the relevance of the topic, popularity in publication categories, and the dynamics of the number of publications over time. The graphs of the datasets analyzed are described and illustrated below.

### 4.1. Web of Science

**Figure 22:**
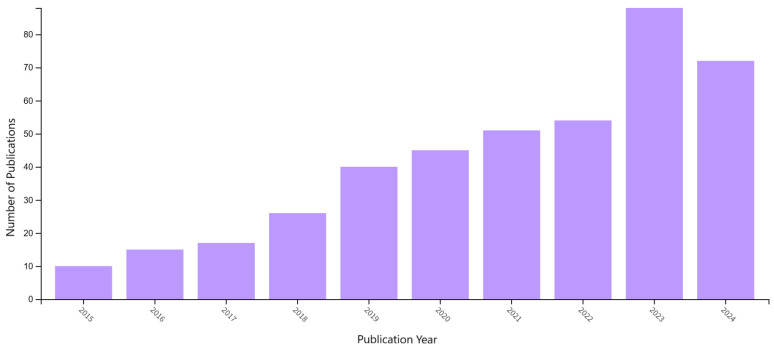
Graphical representation of the analysis of the number of publications from 2015 to 2024 in the Web of Science database.

In accordance with the established criteria, 418 publications of various types were published during this period. Data from the search and analysis of publications indicate a continuous increase in the number of publications over time, which reflects the growing popularity, relevance, and interest in this topic (see Figure 22). In the year 2024, an extreme decline in the number of publications in this database is noticeable. This upward trend is primarily due to the improved availability of new materials and the expanding opportunities for research in the field of materials engineering.

**Figure 23:**
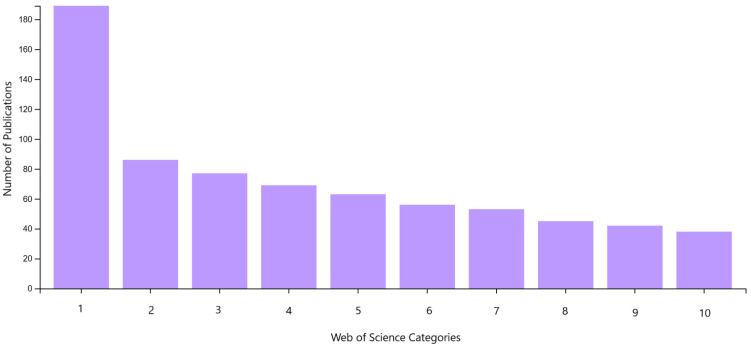
Graphical representation of the analysis of the number of publications from 2015 to 2024 by category in the Web of Science database. (1) Materials Science Multidisciplinary; (2) Nanoscience Nanotechnology; (3) Robotics; (4) Polymer Science; (5) Chemistry Multidisciplinary; (6) Physics Applied; (7) Chemistry Physical; (8) Engineering Electrical Electronic; (9) Instruments Instrumentation; (10) Physics Condensed Matter.

The largest number of publications were in the following categories: Materials Science, Multidisciplinary (45.2%); Nanoscience, Nanotechnology (20.6%); Robotics (18.4%); and Polymer Science (16.5%) (see Figure 23). The most popular publication category in the Web of Science database is Materials Science Multidisciplinary, which contains 189 publications.

**Figure 24:**
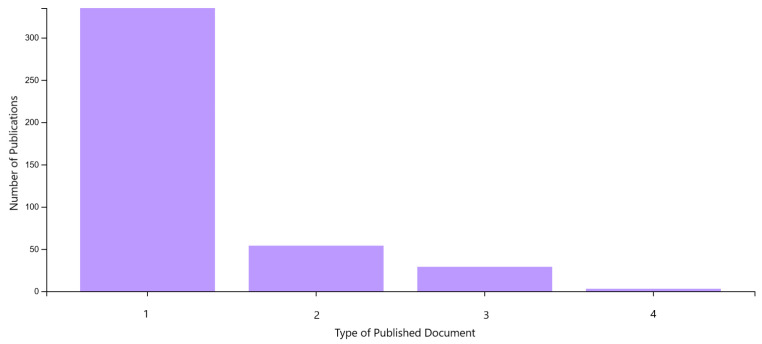
Graphical representation of the analysis of the number of publications from 2014 to 2023 according to the type of published document in the Web of Science database. (1) Article; (2) Proceeding Paper; (3) Review Article; (4) Early Access.

According to the results of the analysis, the largest number of publications in the Web of Science metadata were of the following types: Article (80.1%), Proceeding Paper (12.9%), Review Article (6.9%), and Early Access (0.72%) (see Figure 24). The most frequently published type of document is the Article, with 335 publications.

### 4.2. Scopus

**Figure 25:**
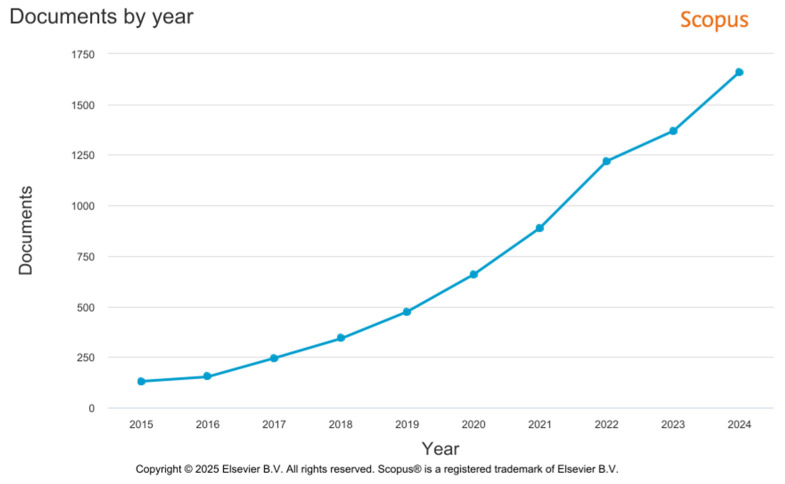
Graphical representation of the analysis of the number of publications from 2014 to 2023 in the Scopus database.

Similarly to the Web of Science metadata analysis, the analysis of the Scopus metadata also shows a dynamic increase in the number of publications from 2015 to 2024 (see Figure 25). According to the established criteria, 7128 documents of various types were published during this period. The main reasons for the increase in published documents include improved availability of new materials and expanded research opportunities in the field of engineering.

**Figure 26:**
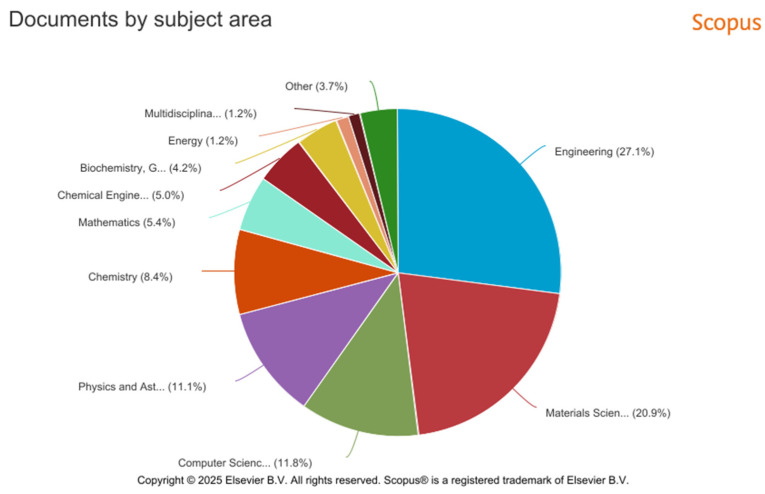
Graphical representation of the analysis of the number of publications from 2014 to 2023 by category in the Scopus database.

The largest number of publications was in the following categories: Engineering (27.1%), Material Science (20.9%), Computer Science (11.8%), and Physics and Astrophysics (11.1%) (see Figure 26). The most popular publication category in the Scopus metadata is Engineering, which includes 4597 publications.

**Figure 27:**
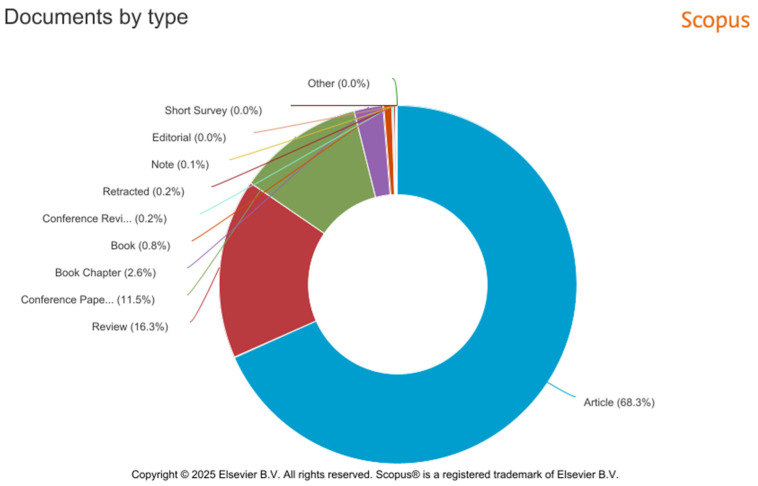
Graphical representation of the analysis of the number of publications from 2014 to 2023 according to the type of published document in the Scopus database.

According to the results of the metadata analysis in the Scopus database, there is a noticeable difference in the types of published documents, with Scopus containing a larger variety of document types according to the keywords (see Figure 27). The most common types of published documents are Article (68.3%, 4871 documents), Review (16.3%, 1160 documents), Conference Paper (11.5%, 818 documents), Book Chapter (2.6%, 188 documents), and Book (0.8%, 54 documents). Articles are the most frequently published document type.

The analysis conducted and described above indicates the relevance of this topic, the continuous growth in the number of publications, and highlights the scientific fields where this issue is most popular.

## 5. Conclusions

The presented article focuses on an overview of the published literature on the selected issue, the structure and mechanical properties of polymers, their implementation in robotics, and an analysis of the topic’s relevance. This concise review provides a comprehensive and detailed overview of the general nature of polymers, their mechanical, physical, and chemical properties, and their potential applications in robotics. The primary focus of the review is on materials such as SMPs, thermoplastic polymers, covalently crosslinked polymers, and magnetic shape memory polymers, which can be used in handling robots, particularly in the construction of gripping mechanisms.

The relevance of the topic is further confirmed by a bibliometric analysis conducted using two multidisciplinary databases—Web of Science and Scopus. The analysis focused on publications from the last decade containing keywords such as gripper, soft, and polymer. Results indicate a steady and significant increase in the number of publications, highlighting the growing interest and scientific activity in this field. Scientific articles dominate the publication types (80.1% in Web of Science and 68.3% in Scopus), with the majority coming from the fields of Engineering (27.1%) and Multidisciplinary Materials Science (45.2%).

Key findings show that polymers, especially SMPs, are increasingly integrated into soft robotic systems due to their flexibility, responsiveness to stimuli, and lightweight structure. Statistical data supports a growing trend toward smart material use in robotics, with applications expanding beyond traditional manufacturing into medical devices, rehabilitation, precision gripping, and adaptive wearables. From a market perspective, three main application areas stand out:

- Biomedical technologies—including soft surgical tools, prosthetics, and drug delivery systems.
- Industrial automation—such as precision gripping and adaptive manipulation in assembly lines.
- Wearable robotics and assistive devices—utilizing the softness and responsiveness of polymers for user comfort and safety.

Future technological opportunities lie in the integration of SMPs with additive manufacturing (3D/4D printing), multi-stimuli-responsive systems, and reconfigurable structures. The development of hybrid materials, smart actuation mechanisms, and programmable architectures will play a crucial role in extending the practical use of soft robotics in various sectors.

Overall, the continuous advancement in polymer science and soft robotics suggests a strong interdisciplinary synergy, where innovations in material design directly contribute to the development of safer, smarter, and more adaptable robotic systems.

## Data Availability

Not applicable.
